# Moderate Intra-Group Bias Maximizes Cooperation on Interdependent Populations

**DOI:** 10.1371/journal.pone.0088412

**Published:** 2014-02-12

**Authors:** Changbing Tang, Zhen Wang, Xiang Li

**Affiliations:** 1 Adaptive Networks and Control Lab, Department of Electronic Engineering, Fudan University, Shanghai, PR China; 2 Department of Physics, Hong Kong Baptist University, Kowloon Tong, Hong Kong; 3 Center for Nonlinear Studies and the Beijing-Hong Kong-Singapore Joint Center for Nonlinear and Complex systems (Hong Kong), Hong Kong Baptist University, Kowloon Tong, Hong Kong; University of Maribor, Slovenia

## Abstract

Evolutionary game theory on spatial structures has received increasing attention during the past decades. However, the majority of these achievements focuses on single and static population structures, which is not fully consistent with the fact that real structures are composed of many interactive groups. These groups are interdependent on each other and present dynamical features, in which individuals mimic the strategy of neighbors and switch their partnerships continually. It is however unclear how the dynamical and interdependent interactions among groups affect the evolution of collective behaviors. In this work, we employ the prisoner's dilemma game to investigate how the dynamics of structure influences cooperation on interdependent populations, where populations are represented by group structures. It is found that the more robust the links between cooperators (or the more fragile the links between cooperators and defectors), the more prevalent of cooperation. Furthermore, theoretical analysis shows that the intra-group bias can favor cooperation, which is only possible when individuals are likely to attach neighbors within the same group. Yet, interestingly, cooperation can be even inhibited for large intra-group bias, allowing the moderate intra-group bias maximizes the cooperation level.

## Introduction

Cooperation is a widely observed phenomenon in social science, biology and economics [1], [2]. However, cooperative behavior apparently contradicts the natural selection [3]: Selfish players always have a higher average fitness than that of cooperators, since selfish players enjoy the benefits from the cooperation of others without associated costs. Therefore, it has fascinated many interests from natural and social scientists to understand the emergence and the stability of cooperation.

Within the interdisciplinary field of evolutionary game theory, this puzzle benefits from techniques of biology, economy, computer sciences, and physics [4], [5]. As a metaphor, the prisoner's dilemma (PD) game has attracted great attention in both theoretical and experimental studies to investigate the evolution of cooperation [6]–[12]. In a typical PD game, two players simultaneously decide whether they act as a cooperator (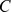) or a defector (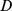). 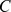
s are willing to engage in cooperative tasks, while 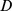
s prefer not to. They will receive the reward, 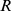, if both cooperate, and the punishment, 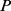, if both defect. However, if one player defects while the other decides to cooperate, the former will get the temptation, 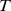, while the latter will get the sucker's payoff, 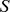. Namely, the local interaction between 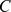 and 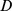 is given by the following payoff matrix:
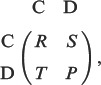
(1)where these payoffs satisfy 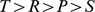 and 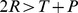. It is obvious that players prefer to defect if they wish to maximize their own payoffs, whatever the opponent's decision.

Various mechanisms to support the evolution of cooperation have been identified recently [13]–[18], such as direct reciprocity, indirect reciprocity, group selection and network reciprocity [19]. The most famous context is spatial games introduced by Nowak and May [20], where players are arranged on a spatially structured population and interact with their neighbors only. It is shown that cooperators could survive by means of forming compact clusters, which minimize the exploitation of defectors. In the line of this seminal achievement, the role of spatial game and its underlying promoted mechanisms in evolutionary games have been intensively explored, such as the mobility of players [21]–[23], different evolutionary time scales [24]–[27], social diversity [28], [29], heterogeneous ability and aspiration [30], [31] (for comprehensive reviews refer to Ref. [32]).

Though large amounts of work upon spatial reciprocity are available, the main attention remains in an isolated and single structure. In human societies, empirical evidences have shown that the realistic structures are composed of many interactive groups, which interact with each other over time [33]–[40]. In this context, the evolution behavior traits have been considered underlying the interdependent populations, where populations are represented by group structures to account for different social types. Note that this framework is similar with previous studies that have addressed the structure on interdependent networks, in the sense that the success of one node in a given group not only depends on the nodes in the same group, but also replies on the states of other nodes in other groups. Taking some examples more specifically, in a recent paper [41], where the biased utility function on interdependent networks were implemented, it was shown that the stronger the bias in the utility function, the higher the level of public cooperation. While in [42], a replicator such as evolutionary game dynamics took place on interdependent populations, cooperative behaviors are fixed on the system (even if the system is well-mixed). Moreover, it was also a remarkable hint that only an intermediate density of sufficiently strong interactions between groups could lead the optimal resolution of social dilemmas [43], [44].

Aside from the effect of spatial structure and its various promoted mechanisms, the co-evolution of game models also attracts numerous attention [45]–[52], which not only reflects the evolving of strategies over time, but also characterizes the adaptive development of topologies and/or update rules. In particular, the interdependent populations in our real social life are dynamical and changing over time. Besides, the essence of evolutionary game theory on interdependent populations remains unclear, especially for the question how the structure of dynamical and interdependent populations affects the evolution of cooperation. Therefore, we introduce an intra-group bias based rewiring probability, and focus on co-evolution of strategy and structure to investigate the evolution of cooperation on interdependent populations. Within the fast rewiring process, we derive a simple rule quantitatively revealing how the link breaking probability and intra-group bias are chosen to stabilize cooperation. Interestingly, though cooperation is favored by intra-group bias conditionally, it is precluded for a large intra-group bias, which uncovers that the moderate intra-group bias maximizes the cooperation level.

## Model and Analysis

### 2.1 Model

We consider the co-evolution of strategy and topology structure of the PD game. Each player can be one of the two strategies, either cooperation 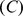 or defection 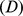, where 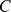
s incur a cost 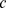 and provide a benefit 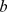 to its opponent (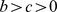), while 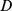
s neither incur costs nor provide benefits. The local interaction between 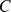 and 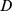 is given by the payoff matrix 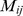, which is a simplified version of Eq. (1):
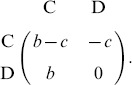
(2)


**Figure 1:**
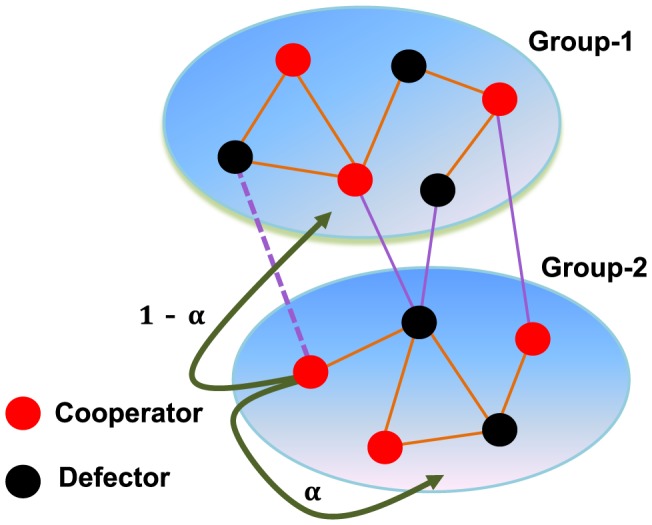
The schematic presentation for the linking adjustment between interdependent populations. Two types of players, 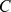 and 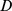, are arranged on the nodes of two interactive groups: Group-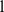 and Group-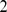, respectively. If the dashed link is selected in the topological evolution, it will be broken off with probability 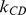. If the dashed link is broken, one of the two players (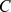 or 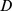) occupying the two extremes of the broken link is selected randomly. Subsequently, the selected player (marked by red circle) switches to another player who is not its current neighbor: it will choose the player in its own group with probability 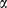, and choose the player in the other group with probability 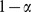.

Initially, the whole population is located at interdependent populations, which consists of Group-1 and Group-2 (see Fig. 1). Each Group-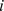 (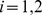) is represented by a network structure with size 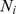, leading to the size of total population 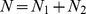. The average degree of Group-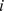 is 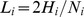, where 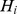 is the total number of links in Group-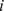. Assume 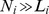, which implies that each player has a limited number of neighbors compared with the population size of the group. Denote the number of links connecting two nodes 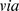 intra-group interactions as 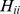 (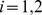), while the number of links connecting two nodes 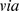 inter-group interactions as 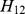 (or 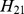). Then, the total number of links is 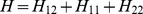.

At each time step, the event of updating strategy takes place with probability 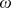, otherwise link adjustment happens with probability 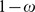. Here, 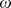 governs the dynamical timescales between strategy updating and topology evolution.

For the strategy updating, we adopt the Fermi dynamics [53], [54] on interdependent populations. Each player is allowed to play with all its current neighbours, and obtains an accumulated payoff. Player 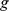 in the whole population is selected at random, subsequently player 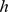 is selected among 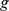
's current neighbors. Then, the strategy of focal player 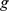 tries to replace that of neighbor 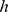 with probability 
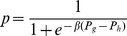
, where 
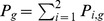
 represents the total payoff of player 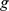 (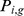 is the payoff of player 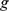 obtained from Group-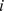). 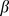 denotes the imitation intensity, measuring how strongly the imitation behavior depends on the payoff difference [55]. In this work, we consider the weak selection (i.e., 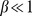), i.e., one phenotype is slightly advantageous, and the effects of payoff differences are small, such that the evolutionary dynamics are mainly driven by random fluctuations.

For the linking dynamics, each link is assigned a label 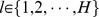 as its name. Assume players will leave or break interactions when they dissatisfy with the current situations. In fact, the social interactions between players in evolve with time based on aspiration payoff [30], [31], reputation [56], [57], and other mechanisms [49], [51]. To characterize the dynamics of structure with various kinds of relationship, we introduce probability 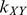 to estimate whether the 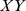-type link is broken. At each time of linking dynamics, link 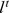 of type 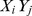 is selected from the whole interdependent populations at random (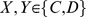, 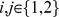). With probability 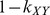, the selected link 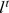 remains unchanged, otherwise, the selected link is broken. If the link is broken, then one node is selected randomly from the two, and it tries to find another partner to connect with. With probability 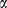, the neighbor is only selected within the same group, otherwise, the potential neighbor is chosen from the other group (see Fig. 1). Here, 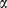 is the intra-group attaching bias between two interactive groups, which reflects the propensity to rewire neighbors via intra-group interactions.

It is worth noting that 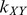 is time-invariant and describes an intrinsic quantity of the linking dynamics. It is shown that the duration time of 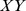 link obeys the geometric distribution with parameter 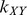 [58], [59]. Therefore, the inverse of 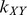 can be taken as the the average interaction rate between 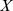 and 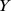. Besides, the total number of links remains constant during the linking dynamics of the interdependent populations as in [24], [47].

### 2.2 Evolutionary dynamics on interdependent populations

Denoted the types of link 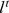 as 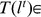 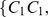 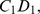 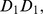 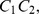 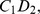 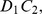 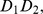 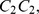 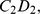 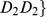. Then, the dynamics of 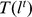 is captured by a Markov chain with transition matrix 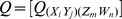, whose entry 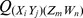 is the transition probability that link 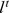 of type 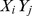 transforms to link 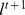 of type 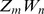. The transition matrix of such a Markov chain is given by 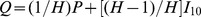 (See Text S1).

Since the Markov chain is irreducible and aperiodic, there exists a unique stationary distribution 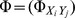 determined by equation 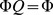 [58],
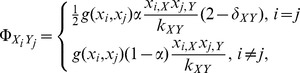
(3)where 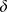 indicates the Kronecker delta, 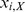 is the frequency of strategy 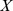 in Group-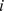, and 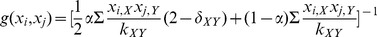 is the normalization factor. The normalized stationary distribution 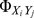 represents the fraction of 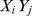 links in the whole population. Therefore, the average number of 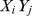 links is 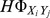.

In the case of a fast rewiring process, i.e., 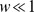, the strategy updating occurs less frequently than linking adjustment [24], [25], and the structure of groups is almost in the stationary state with the distribution described by Eq. (3) when the strategy evolution occurs. In this case, the average fitness function of strategies 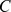 and 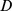 in Group-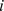 is given by
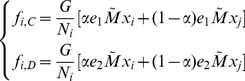
(4)where 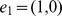, 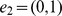, 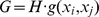, 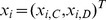, and
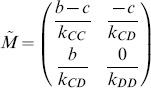
(5)is a modified payoff matrix of Eq. (2), whose payoff entry is rescaled by the inverse of the breaking probability. Note that the first term in the bracket of Eq. (4) represents the payoff obtained 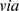 interactions in the same group, while the second term of Eq. (4) represents the payoff obtained 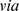 interactions belonging to different groups. Therefore, the payoff of each player in the interdependent populations relies on the neighbors in not only the same group but also the other group.

**Figure 2:**
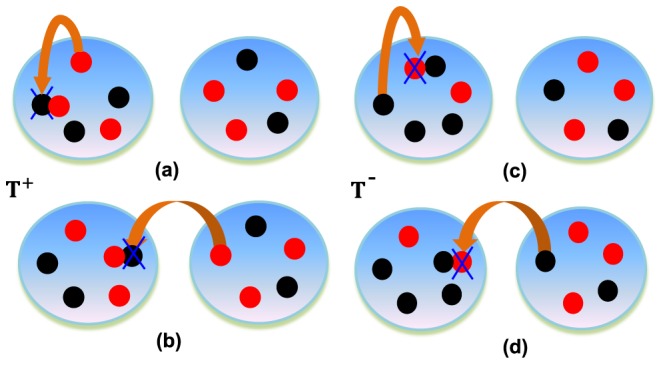
An illustrative transition probability of interdependent populations. The left: probability that the number of 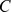 players in Group-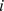 increases from 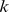 to 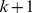 is 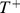, which is caused by a player 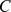 in Group-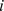 (a) (or Group-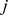 (b)) replacing a player 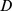 in Group-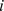. The right: probability that the number of 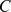 players in Group-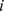 decreases from 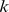 to 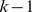 is 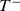, which is caused by a player 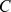 in Group-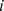 replaced by a player 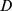 in Group-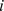 (c) (or Group-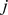 (d)).

Besides, as shown in Fig. 2, the change of 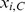 in Group-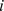 is due to the pairwise comparison between the focal player 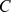 in Group-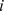 and player 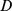 in Group-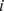 (or Group-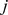), which yields the transition probabilities
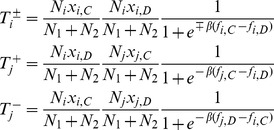
(6)where 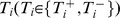 is the transition probability caused by the pairwise comparison occurring in the same group, while 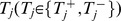 is the transition probability caused by the pairwise comparison occurring in different groups.

For a large population, the stochastic process can be well approximated by a set of stochastic differential equations referring to Langevin dynamics [60]. To the pairwise comparison process occurring on interdependent populations, the Langevin dynamics yields 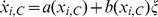, where 
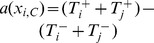
, 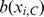 is the effective terms, and 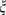 is the uncorrelated Gaussian noise. Since 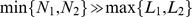, the stochastic term vanishes [61], [62], which leads to
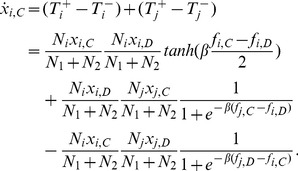
(7)


Especially, when 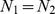, the strategy evolution degenerates to an extension of the replicator dynamics, which yields
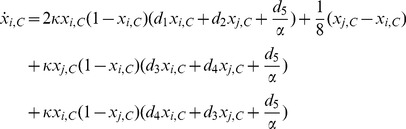
(8)where 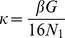 is a constant factor influencing the timescale only. Besides, 
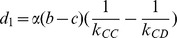
, 
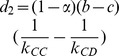
, 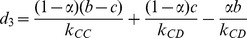, 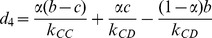, 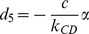. Here, we assume 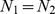 for simplicity without loss of generality, and the numerical results for 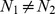 are consistent with this simplification.

Note that the unit square 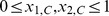 is the invariant set of 2-D plane. From Eq. (8), we obtain three possible equilibria 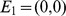, 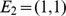, and 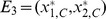 (
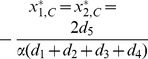
). For such three equilibria, the Jacobian matrix 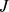 of (8) has the form
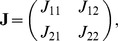
where 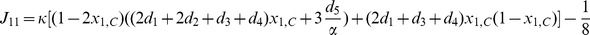, 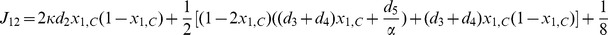, 
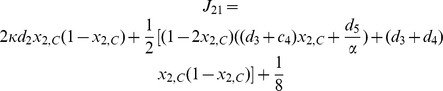
, 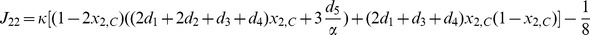. The corresponding eigenvalues of Jacobian matrix 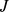 at 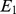, 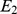 and 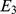 are listed in Table 1.

**Table 1:** Eigenvalues of Jacobin matrix 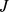 at 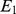, 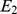 and 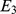.

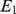	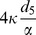	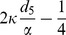
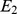	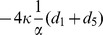	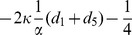
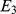	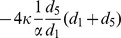	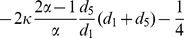

When 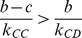, 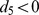 and 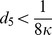, which implies that the eigenvalues of Jacobian matrix 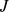 are both negative for 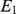; 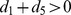 and 
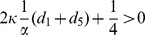
, which implies that both the the eigenvalues of Jacobian matrix 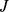 are negative for 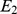. Therefore, both 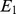 and 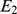 are stable. For the interior equilibrium 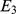, when 
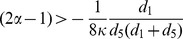
, the eigenvalues of Jacobian matrix 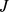 are both positive for 
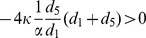
 and 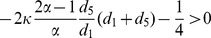, which indicates that the eigenvalues of Jacobian matrix 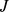 are positive for 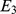. Therefore, when 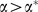 
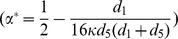
, 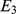 is an unstable equilibrium.

## Results

**Figure 3:**
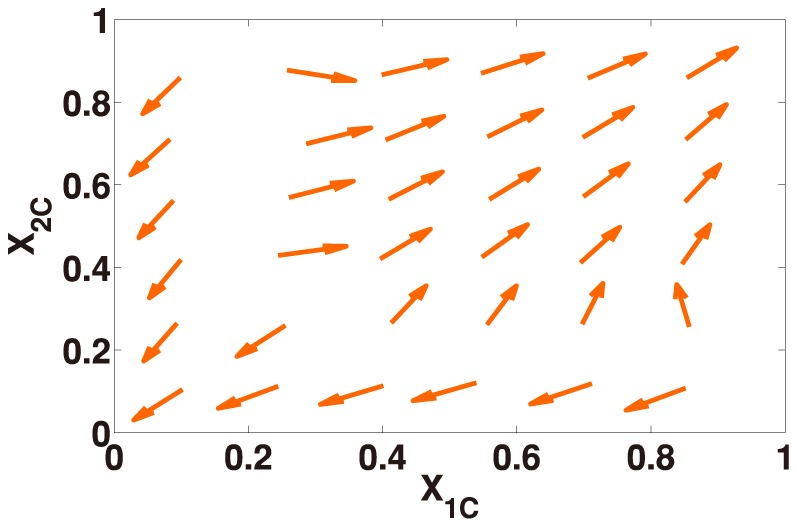
Phase portrait of Eq. (5) under weak selection. The direction of the velocity field is denoted by arrows. We set 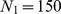, 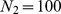, 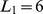, 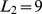, 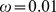, and 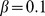. Under the condition 
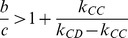
 and 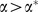 (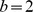, 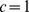, 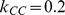, 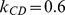, 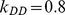 and 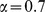), the velocity field converges to the corner equilibrium 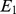 or 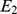 independent of the initialization.

Let us now consider how the co-evolution of strategy and link dynamics affects cooperation on the interdependent populations. When 
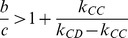
 and 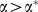 (
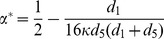
), the eigenvalues of the Jacobian matrix in Eq. (8) are negative for both 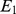 and 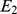, yet positive for 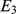. Thus, both 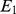 and 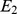 are stable, and 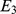 is unstable, which leads to the final state converging to 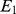 or 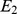. Namely, Eq. (8) in the whole group is composed of all-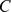 (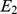) or all-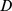 (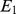) (See Fig. 3). On the other hand, when 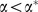, 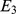 becomes a saddle-point. Therefore, strategies 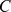 and 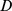 are bistable on the interdependent populations.

**Figure 4:**
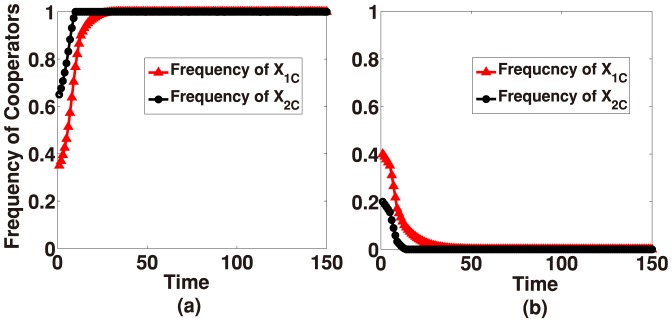
Simultaneous time-evolution of the frequency of cooperator in Group-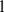 and Group-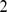 under weak selection. Initially, strategy 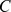 is randomly distributed in Group-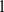 and Group-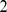. For all plots, we set 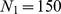, 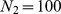, 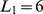, 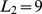, 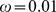, 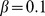, 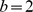, 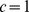, 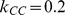, 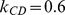, 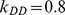 and 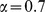. (a) When 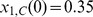 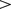 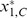 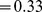 and 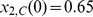 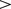 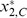 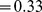, Eq. (5) converges to the state of all-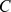 independent of the initialization. (b) When 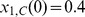 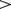 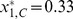, but 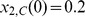 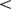 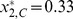, Eq. (5) converges to the state of all-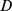.

Besides, the equilibrium 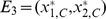 determines the attraction basin of cooperation 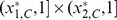. If the initial condition, 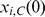, is more than the critical value of 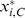 (i.e., 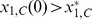 and 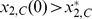), then system (8) converges to all-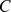; otherwise, it reaches all-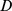. The effect of initialization on the frequency of strategy 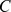 in both groups is shown in Fig. 4. In other words, the PD game with link dynamics corresponds to a coordination game in well-mixed populations, where both cooperation and defection are best replies to themselves [63]. Thus, cooperation is stable only when
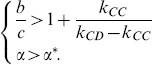
(9)


Note that Eq. (9) is necessary for emerging cooperation, namely, the co-evolution of strategy and link dynamics can favor cooperation if the benefit-to-cost ratio 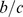 exceeds 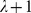 (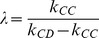). This condition is intuitive: the critical benefit-to-cost ratio is a decreasing function of 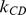 but an increasing function of 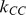. Indeed, the evolution of cooperation is promoted if 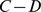 links are more fragile than 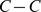 links, which coincides with the results in [24], [64]. Besides, quantity 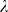 measures the propensity for cooperators to form clusters, and 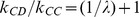 characterizes the fragility ratio between 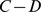 link and 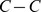 link. Decreasing 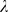 allows cooperators to spread more effectively [22]. In particular, when 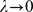, a cooperator is more likely to play with cooperators rather than defectors, and easier to form clusters. In this sense, 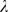 illustrates how likely a cooperator is to interact with a cooperator.

**Figure 5:**
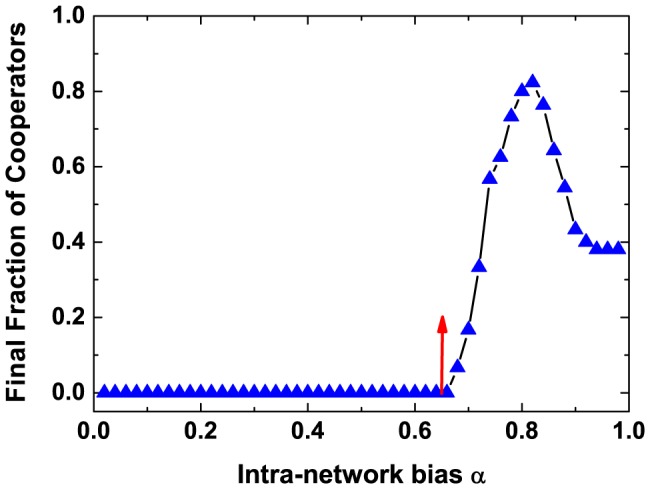
Final fraction of cooperators as a function of the intra-group bias 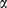. We set 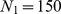, 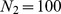, 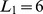, 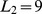, 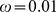, 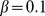, 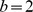, 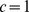, 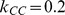, 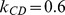 and 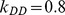. The analytical value of 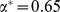, which is marked out by red arrow in the plot. Each data point is averaged over 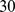 independent runs, and in each realization, we set 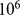 time steps to ensure the evolution of dynamics in steady states. It is shown that there exists a moderate intra-group bias leading to maximum cooperation level in the whole populations.

More interestingly, there exists a lower bound of the intra-group bias between two groups for emerging cooperation. The intra-group bias between two groups hinders the invasion of defectors on the single group, thereby influences the evolution of cooperation. When the value of 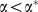, the interior equilibrium becomes a saddle point. Thus, small value of 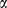 is excluded to the model of coordination game, and the cooperation will never emerges when 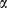 is smaller than the critical value 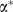. For instance, with a small value of 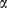, a defector on Group-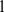 might take advantage from the vicinity of cooperators on Group-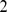, because the corresponding interactions on Group-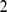 may supply enough resource to be exploited, which results in the prosperous of defection. On the other hand, big intra-group bias also ignores the inter-group interactions between two interdependent groups. In this way, increasing 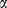 narrows the attraction basin of cooperation 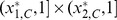, and makes it difficult for cooperation to gain a foothold in the population. Therefore, the intra-group bias favors the cooperation for players are likely to switching to attach neighbors within the same group. However, too large intra-group bias hinders the prosperity of cooperation, allowing the moderate intra-group bias maximizes the cooperation level (See Fig. 5).

**Figure 6:**
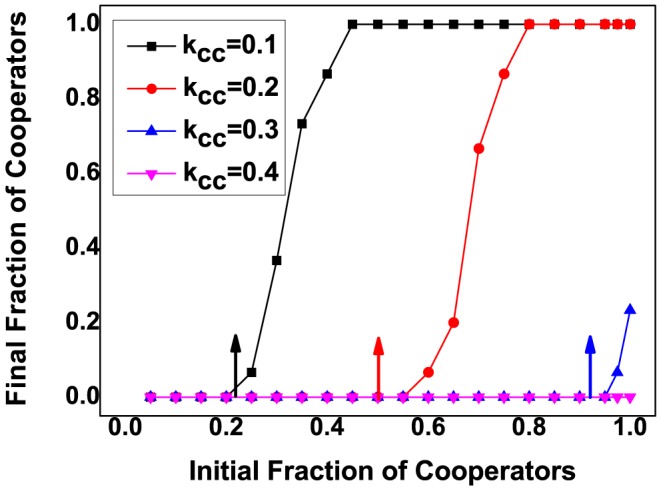
Final fraction of cooperators as a function of initial fraction of cooperators with different 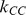. According to Eq. (8), we obtain that when 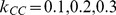, the analytical results of 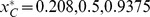 respectively, which are marked out by arrows in the plot. For all the three line in the plot, we set 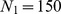, 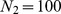, 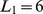, 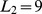, 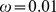, 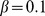, 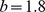, 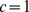, 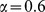, 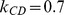 and 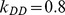. The simulation results show that the initial frequency of cooperators 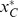 increases with increasing of 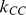. Large 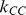 narrows the attraction basin of cooperation, which makes the flourishing of cooperation difficult.

**Figure 7:**
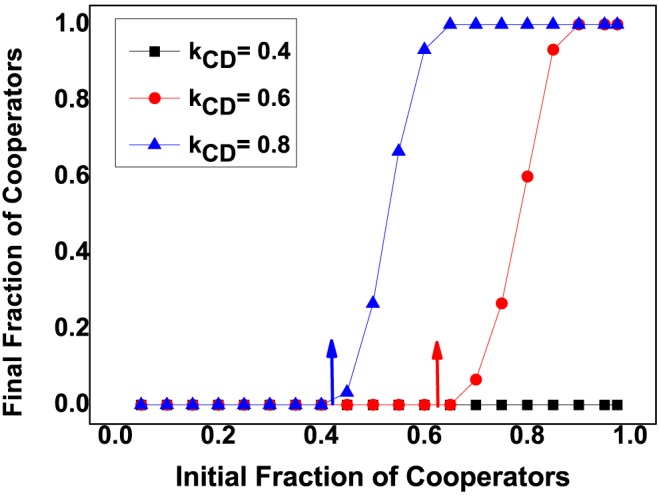
Final fraction of cooperators as a function of initial fraction of cooperators with different 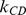. According to Eq. (8), we obtain that when 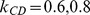, the analytical results of 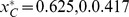 respectively, which are marked out by arrows in the plot. For all the three line in the plot, we set 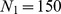, 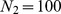, 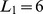, 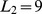, 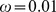, 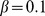, 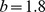, 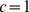, 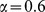, 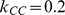 and 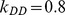. The simulation results show that the initial frequency of cooperators 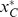 decreases with increasing of 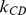, i.e., large 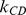 enlarges the attraction basin of cooperation and promotes the flourishing of cooperation.

Till now, we have shown a simple rule telling how cooperation emerges with linking dynamics. Although, condition (9) guarantees the necessity of emerging cooperation, it's not sufficient to make cooperation advantageous. To make cooperators gain a foothold in the population of coordination game, the initial frequency of cooperators in the whole group should exceeds the unstable interior fixed point, which equals
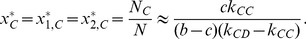
(10)Similarly, 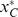 is a decreasing function of 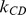 and an increasing function of 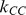. Thus, decreasing 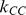 and increasing 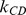 enlarges the attraction basin of cooperation 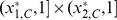, and makes it easier for cooperation thrives. Fig. 6 shows that the critical value of unstable interior fixed point 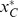 increases with increasing 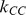, i.e. a larger 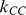 leads to the larger 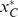, which makes the flourishing of cooperation more difficult. Increasing the value of 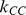 to 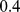, cooperators are never advantageous compared to defectors. Thus, cooperators are never favored by selection. Besides, the critical unstable interior fixed point 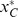 decreases with increase of 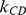 (see Fig. 7), i.e., a larger 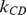 leads to the smaller 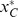, which is beneficial to the flourishing of cooperation. Contrarily, a smaller 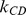 prevents the flourishing of cooperation. Specially, when decreases 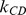 to 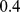, cooperators are never advantageous compared to defectors.

## Conclusions

To sum up, we have established a microscopic model on the co-evolutionary dynamics of cooperation and interdependent populations. Under the assumption of fast structure evolving, we analytically arrived in the macro-dynamics at the population level: an extended replicator equation which incorporates both the interactions of groups and the strategy evolution. Based on this extended equation, it is shown that the less the fragile cooperator-cooperator links (or the more the fragile cooperator-defector links), the easier the emergence of cooperation. This result is consistent with previous findings that assortments of cooperators are likely to invade a defector population and escape from the exploitation of defector mutants [24], [25], [47], which paves the way for both emergence and stabilization of cooperation.

Interestingly, we have revealed that the dynamical interactions on interdependent populations can greatly affect the evolution of cooperation: cooperation can only emerge when intra-group bias is big enough. This is intuitive in the sense that intra-group bias can lead to cooperation [35], [36], which indicates that it might be more likely to establish neighbors within the same group. However, counterintuitive results also arise: it is unlikely that the more possible individuals establishing neighbors within the same group, the higher cooperation level is. In fact, too large intra-group switching bias inhibits cooperation [42], [43]. This reminds us with the migration effect: Neither too large nor too small mutation rate benefits cooperation. Thus, it would be beneficial for cooperators to move from time to time in order to hunt/establish a paradise to live, since this accidental moving can help the cooperators to escape from the nasty environment consisting of mainly defectors. Yet large migration rates make the population approximately well-mixed destroying the cooperation clustering, which deters cooperation. The switching rate of attaching neighbors outside the group can be viewed as the migration rate, thus leads to moderate switching rate maximizing cooperation. Our work shed light on how the dynamic of interdependent have an impact on the cooperation. This insight might also be constructive to other collective behaviors such as swarming and coordination and opinion formation. Works along those lines are in progress.

## Supporting Information

Text S1 **Embedded Markov chain approximation for linking dynamics.**
(PDF)
