# Supplementary material for: Moderate Intra-Group Bias Maximizes Cooperation on Interdependent Populations
Source: PLoS One. 2014 Feb 12;9(2):e88412. doi: 10.1371/journal.pone.0088412 (PMC3922813; doi:10.1371/journal.pone.0088412)
Supplement: Text S1 — Embedded Markov chain approximation for linking dynamics. (PDF) [file pone.0088412.s001.pdf]

Text S1

Embedded Markov chain approximation for linking dynamics

It is impossible that the original link and the transformed one share no identical extreme in our linking dynamics, and the entries of the transition matrix must be zero if $\{X_i, Y_j\} \bigcap \{Z_m, W_n\} = \emptyset$. Once the specified link is selected, we define the conditional transition matrix P. Otherwise, the link is not selected and its type keeps constant, the conditional transition matrix is the identity matrix I_{10}. Therefore, the transition matrix of such a Markov chain is given by

$$Q = (1/H)P + [(H-1)/H]I_{10}, \tag{1}$$

where P is the conditional transition matrix, and its entry $P_{(X_iY_j)(Z_mW_n)}$ indicates the transition probability with which a link of type X_iY_j transforms to a link of type Z_mW_n. Furthermore, the conditional transition matrix P is given by

$$\begin{pmatrix} P_{11} & P_{12} & P_{13} \\ P_{21} & P_{22} & P_{23} \\ P_{31} & P_{32} & P_{33} \end{pmatrix},$$

where $P_{13} = P_{31}$ is the zero matrix, and

$$P_{11} = \begin{pmatrix} (1-k_{CC}) + \alpha k_{CC}x_{1,C} & \alpha k_{CC}x_{1,D} & 0 \\ \frac{1}{2}\alpha k_{CD}x_{1,C} & (1-k_{CD}) + \frac{1}{2}\alpha k_{CD} & \frac{1}{2}\alpha k_{CD}x_{1,D} \\ 0 & \alpha k_{DD}x_{1,C} & (1-k_{DD}) + \alpha k_{DD}x_{1,D} \end{pmatrix},$$

$$P_{12} = \begin{pmatrix} (1-\alpha)k_{CC}x_{2,C} & (1-\alpha)k_{CC}x_{2,D} & 0 & 0 \\ \frac{1}{2}(1-\alpha)k_{CD}x_{2,C} & \frac{1}{2}(1-\alpha)k_{CD}x_{2,D} & \frac{1}{2}(1-\alpha)k_{CD}x_{2,C} & \frac{1}{2}(1-\alpha)k_{CD}x_{2,D} \\ 0 & 0 & (1-\alpha)k_{DD}x_{2,C} & (1-\alpha)k_{DD}x_{2,D} \end{pmatrix},$$

$$P_{21} = \begin{pmatrix} \frac{1}{2}\alpha k_{CC}x_{1,C} & \frac{1}{2}\alpha k_{CC}x_{1,D} & 0 \\ \frac{1}{2}\alpha k_{CD}x_{1,C} & \frac{1}{2}\alpha k_{CD}x_{1,D} & 0 \\ 0 & \frac{1}{2}\alpha k_{CD}x_{1,C} & \frac{1}{2}\alpha k_{CD}x_{1,D} \\ 0 & \frac{1}{2}\alpha k_{DD}x_{1,C} & \frac{1}{2}\alpha k_{DD}x_{1,D} \end{pmatrix}, P_{23} = \begin{pmatrix} \frac{1}{2}\alpha k_{CC}x_{2,C} & \frac{1}{2}\alpha k_{CC}x_{2,D} & 0 \\ 0 & \frac{1}{2}\alpha k_{CD}x_{2,C} & \frac{1}{2}\alpha k_{CD}x_{2,D} \\ \frac{1}{2}\alpha k_{CD}x_{2,C} & \frac{1}{2}\alpha k_{CD}x_{2,D} & 0 \\ 0 & \frac{1}{2}\alpha k_{DD}x_{2,C} & \frac{1}{2}\alpha k_{DD}x_{2,D} \end{pmatrix},$$

$$P_{22} = \begin{pmatrix} (1-k_{CC}) + \frac{(1-\alpha)k_{CC}}{2} & \frac{1-\alpha}{2}k_{CC}x_{2,D} & \frac{1-\alpha}{2}k_{CC}x_{1,D} & 0 \\ \frac{1-\alpha}{2}k_{CD}x_{2,C} & (1-k_{CD}) + \frac{(1-\alpha)k_{CD}}{2} & 0 & \frac{1-\alpha}{2}k_{CD}x_{1,D} \\ \frac{1-\alpha}{2}k_{CD}x_{1,C} & 0 & (1-k_{CD}) + \frac{(1-\alpha)k_{CD}}{2} & \frac{1-\alpha}{2}k_{CD}x_{2,D} \\ 0 & \frac{1-\alpha}{2}k_{DD}x_{1,C} & \frac{1-\alpha}{2}k_{DD}x_{2,C} & (1-k_{DD}) + \frac{(1-\alpha)k_{DD}}{2} \end{pmatrix},$$

$$P_{32} = \begin{pmatrix} (1-\alpha)k_{CC}x_{1,C} & 0 & (1-\alpha)k_{CC}x_{1,D} & 0 \\ \frac{1-\alpha}{2}k_{CD}x_{1,C} & \frac{1-\alpha}{2})k_{CD}x_{1,C} & & \\ frac1-\alpha 2k_{CD}x_{1,D} & \frac{1-\alpha}{2}k_{CD}x_{1,D} & & \\ 0 & (1-\alpha)k_{DD}x_{1,C} & 0 & (1-\alpha)k_{DD}x_{1,D} \end{pmatrix},$$

$$P_{33} = \begin{pmatrix} (1-k_{CC}) + \alpha k_{CC}x_{2,C} & \alpha k_{CC}x_{2,D} & 0 \\ \frac{1}{2}\alpha k_{CD}x_{2,C} & (1-k_{CD}) + \frac{1}{2}\alpha k_{CD} & \frac{1}{2}\alpha k_{CD}x_{2,D} \\ 0 & \alpha k_{DD}x_{2,C} & (1-k_{DD}) + \alpha k_{DD}x_{2,D} \end{pmatrix}.$$

Here, $x_{i,X} = N_{i,X}/N_i$ ($i = 1, 2,\ X = C, D$) is the frequency of strategy X in Group-i ($N_{i,X}$ is the number of players performing strategy X on Group-i).

Take $Q_{(C_1D_2)(C_1D_2)} = [2(1-k_{CD}) + (1-\alpha)k_{CD}(x_{1,C} + x_{2,D})]/(2H) + (H-1)/H$ as an example. Link l^t of type C_1D_2 transforms to l^{t+1} of type C_1D_2 in the following cases:
(i) when l^t is not selected in the linking dynamics (with probability $(H-1)/H$);
(ii) when l^t is selected (with probability $1/H$). This happens either when the original C_1D_2 link is not broken (with probability $1-k_{CD}$), or it does break (with probability k_{CD}). In the latter case, the node with strategy C is selected (with probability $1/2$), and it switches to a strategy-D player in Group-2 (with probability $(1-\alpha)x_{2,D}$), or the node with strategy D is selected (with probability $1/2$), and it switches to a strategy-C player in Group-1 (with probability $(1-\alpha)x_{1,C}$).
